# Neoadjuvant camrelizumab plus chemotherapy in locally advanced oesophageal squamous cell carcinoma: a retrospective cohort study

**DOI:** 10.1186/s12893-023-02023-5

**Published:** 2023-05-09

**Authors:** Rui-Qin Zhou, Jun Luo, Lin-Jun Li, Ming Du, Qing-Chen Wu

**Affiliations:** Department of Cardiothoracic Surgery, the First Affiliated Hospital of Chongqing Medical University, Chongqing, 400016 China

**Keywords:** Oesophageal squamous cell carcinoma (ESCC), Neoadjuvant chemotherapy, Immunotherapy, Camrelizumab, Docetaxel, Nedaplatin

## Abstract

**Background:**

Neoadjuvant therapy is recommended to improve the prognosis of oesophageal squamous cell carcinoma (ESCC). As a PD-1 inhibitor developed in China, camrelizumab is more accessible and available for Chinese ESCC patients. Camrelizumab plus neoadjuvant chemotherapy has shown promising efficacy with acceptable toxicity for resectable ESCC in the NIC-ESCC2019 trial. However, this was a single-arm trial, so we conducted a retrospective cohort study to compare neoadjuvant camrelizumab plus chemotherapy with neoadjuvant chemotherapy alone in terms of the safety and efficacy in patients with locally advanced ESCC.

**Methods:**

Between January 2017 and December 2021, patients with stage II–IVa ESCC who received neoadjuvant therapy at the First Affiliated Hospital of Chongqing Medical University and underwent radical oesophagectomy were enrolled in our study. These included 19 patients who received neoadjuvant chemotherapy plus camrelizumab (group 1) and 40 patients who only received neoadjuvant chemotherapy (group 2).

**Results:**

The baseline characteristics of the patients were comparable between the two groups. The pathological complete response (pCR) rate in group 1 was significantly higher than that in group 2 (26.3% vs. 2.5%, *P* = 0.018). All patients in group 1 achieved complete resection (R0), compared with 39 (97.5%) patients in group 2. Adverse events occurred in 16 (84%) patients in group 1 versus 35 (87.5%) patients in group 2. No grade ≥ 4 adverse events occurred in either group. No significant difference was found in surgical outcomes or postoperative complications. The 90-day mortality rate was comparable between the two groups (1 patient died in group 1 versus 2 patients in group 2).

**Conclusions:**

Neoadjuvant camrelizumab plus chemotherapy followed by surgery was associated with a promising pCR rate and a manageable safety profile for patients with locally advanced ESCC.

## Introduction

Oesophageal squamous cell carcinoma (ESCC) is a malignant cancer with a poor prognosis. A multidisciplinary approach is recommended for these patients to improve treatment outcomes. Radical oesophagectomy is considered the most promising treatment strategy for patients with locally advanced ESCC, but the 5-year overall survival (OS) rate remains low. Therefore, neoadjuvant therapy is recommended for locally advanced ESCC, as it can lead to tumour downstaging, a higher rate of R0 resection, and survival benefits in patients who achieve pathological complete response (pCR). In some studies, neoadjuvant chemoradiotherapy (nCRT) showed better histopathologic outcomes than neoadjuvant chemotherapy (nCT) but a similar safety profile. However, there is no clear consensus regarding whether nCRT can increase overall survival [1–3]. There is insufficient evidence to indicate the comparative advantage of nCRT over nCT for ESCC [4]. Hence, exploration of new drugs and the development of better combination treatment strategies are key to improve the prognosis of ESCC.

The combination of chemotherapy with ICIs, such as pembrolizumab and camrelizumab, has been approved as first-line therapy for advanced or metastatic oesophageal carcinoma according to the findings of the Keynote-590 trial and ESCORT-1st trial [5, 6]. As a PD-1 inhibitor developed in China, camrelizumab has exhibited proven effectiveness for ESCC patients. Moreover, it is one-tenth the price of imported PD-1 inhibitors. More ESCC patients have access to this regimen and benefit from it. Camrelizumab plus neoadjuvant chemotherapy also showed promising efficacy with acceptable toxicity for resectable ESCC in the NIC-ESCC2019 trial [7]. However, this was a single-arm trial, so we conducted a retrospective cohort study to compare neoadjuvant camrelizumab plus chemotherapy with neoadjuvant chemotherapy alone in terms of safety and efficacy in patients with locally advanced ESCC. Furthermore, in this study, all surgeries after neoadjuvant therapy were performed by the same experienced surgeon in our department and were the same surgery type of McKeown oesophagectomy and two-field lymphadenectomy, which to some extent makes the surgical outcomes more comparable.

## Patients and methods

### Patients

All patients with locally advanced ESCC who received neoadjuvant therapy at the First Affiliated Hospital of Chongqing Medical University between January 2017 and December 2021 were evaluated in this study. Basic information pertaining to diagnosis and treatment was collected from electronic medical records. All included patients had undergone radical oesophagectomy performed by the same experienced surgeon in our department. Patients who met the following inclusion criteria were enrolled: (1) histological diagnosis of thoracic ESCC clinically staged as T1N1-3M0 or T2-4aN0-3M0; (2) age range, 18–75 years; and (3) Eastern Cooperative Oncology Group (ECOG) performance status ≤ 2. The exclusion criteria were as follows: (1) previous neoadjuvant chemoradiotherapy; (2) serious organ dysfunction or immunodeficiency; (3) metastatic cervical or supraclavicular lymph node; and (4) history of previous gastrectomy or presence of concurrent cancers.

### Pretreatment clinical evaluation and staging

All patients underwent the following investigations before treatment: routine laboratory tests, upper gastrointestinal endoscopy with biopsy, upper gastrointestinal radiography, contrast-enhanced computed tomography (CT) of the neck, thorax and abdomen, brain magnetic resonance imaging (MRI), and emission computed tomography (ECT). Positron emission tomography/CT (PET/CT) was not performed for every patients. Tumours were staged according to the Union for International Cancer Control TNM Classification 8th Edition.

### Neoadjuvant therapy

Patients who received neoadjuvant chemotherapy in combination with camrelizumab (group 1) received 2 cycles of docetaxel (75 mg/m^2^) and nedaplatin (75 mg/m^2^) and were simultaneously administered 2 doses of intravenous camrelizumab (dose: 200 mg every 3 weeks). Patients who received neoadjuvant chemotherapy alone (group 2) received 2 cycles of docetaxel (75 mg/m^2^) and nedaplatin (75 mg/m^2^). The decision to use camrelizumab depended on the patient. Written informed consent for the chosen treatment was obtained from all patients. The toxicity of neoadjuvant therapy was monitored according to the National Cancer Institute Common Terminology Criteria for Adverse Events version 5.0.

### Preoperative clinical assessment

Approximately 3–4 weeks after the completion of neoadjuvant therapy, patients underwent clinical restaging based on the findings of the neck, thoracic and abdominal contrast-enhanced CT scans. Changes in tumour size were evaluated in accordance with the Response Evaluation Criteria in Solid Tumors (RECIST) version 1.1. Patients also underwent physical examinations, routine laboratory tests, echocardiography, and pulmonary function tests to evaluate their suitability for surgery.

### Surgery

Surgery was performed 6–8 weeks after the completion of neoadjuvant therapy. Minimally invasive oesophagectomy via thoracoscopy and laparoscopy was performed by an experienced surgeon in our department. The type of surgical technique was McKeown oesophagectomy with two-field lymphadenectomy; oesophageal reconstruction was performed using a stomach conduit and cervical anastomosis. The length of hospital stay after the operation, surgical outcomes and postoperative complications were recorded. Short-term surgical outcomes, including postoperative 30-day mortality and 90-day mortality, were obtained from treatment records or by telephone follow-up.

### Pathological analysis

The effectiveness of neoadjuvant therapy was evaluated based on the pathological examination reports. The reports included the description of the tumour type and tumour extent, resection margin status, and lymph node status (site, number of resected lymph nodes, and metastatic nodes). Pathological complete response (pCR) was defined as no residual tumour cells in the surgical specimens from the primary site or the resected lymph nodes. R0 resection was defined as no residual tumour tissue. R1 resection was defined as microscopic residual tumour tissue at the resection margin.

### Statistical analysis

Normally distributed continuous variables are presented as the mean ± standard deviation (SD), and nonnormally distributed continuous variables are presented as the median and interquartile range (IQR). Categorical variables are described as frequencies and percentages. Independent-sample *t tests* or Mann‒Whitney U tests were used to compare continuous variables. The chi-square test was used to compare categorical variables. Two-sided *P* values < 0.05 were considered indicative of a significant difference. Statistical analysis was performed using SPSS 26.0 for Windows (IBM Corp).

## Results

### Patient characteristics

Fifty-nine patients were included in the analysis. Among these, 19 patients received neoadjuvant chemotherapy combined with camrelizumab (group 1), and 40 patients received neoadjuvant chemotherapy alone (group 2). Two patients who received neoadjuvant chemotherapy combined with camrelizumab followed by surgery were excluded from the analysis because they had previously received neoadjuvant therapy at other hospitals and because no detailed information on their adverse events could be obtained. The baseline characteristics of the patients are summarized in Table 1. The proportion of patients staged as cT4 was higher in group 1 than in group 2 [14 (73.7%) vs. 28 (70%)], and the proportion of patients staged as cN0 was lower in group 1 than in group 2 [12 (63.2%) vs. 28 (70%)], but the overall clinical T stage and clinical N stage were not significantly different between groups (P = 0.734 and P = 0.215, respectively). The demographics, characteristics and comorbidity status were similar between the two groups. Tumour location and clinical TNM stage were also not significantly different between the two groups.

**Table 1:** Baseline characteristics of the study population

Baseline	Group 1 (n = 19)	Group 2 (n = 40)	*P* value
Age (years)	65.89 ± 6.06	64.50 ± 4.54	0.327
Sex			0.456
Male, n (%)	17 (89.5)	31 (77.5)	
Female, n (%)	2 (10.5)	9 (22.5)	
BMI, kg/m^2^	23.06 ± 2.87	22.60 ± 3.35	0.609
Tumour location, n (%)			0.618
Proximal third	1 (5.3)	6 (15.0)	
Middle third	10 (52.6)	21 (52.5)	
Distal third	8 (42.1)	13 (32.5)	
Comorbidity, n (%)			
Hypertension	6 (31.6)	7 (17.5)	0.377
Diabetes	3 (15.8)	4 (10.0)	0.832
Coronary heart disease	1 (5.3)	2 (5.0)	> 0.999
COPD	6 (31.6)	11 (27.5)	0.747
Clinical T stage, n (%)			0.734
cT1	0	0	
cT2	0	1 (2.5)	
cT3	5 (26.3)	11 (27.5)	
cT4	14 (73.7)	28 (70)	
Clinical N stage, n (%)			0.215
cN0	12 (63.2)	31 (77.5)	
cN1	6 (31.6)	9 (22.5)	
cN2	1 (5.3)	0	
cN3	0	0	
Clinical TNM stage, n (%)			0.534
II	1(5.3)	9(22.5)	
III	4(21.1)	3(7.5)	
IVA	14(73.7)	28(70)	

BMI, body mass index; COPD, chronic obstructive pulmonary disease

## Safety of neoadjuvant therapy and treatment response

The adverse events in the two groups are shown in Table 2. Adverse events occurred in 16 (84%) patients in group 1 versus 35 (87.5%) patients in group 2. No grade ≥ 4 adverse events were observed in either group. Leukopenia [6 (31.6%) vs. 14 (35.0%)] and anorexia [5 (26.3%) vs. 9 (22.5%)] were the most common AEs. Four (21.1%) patients in group 1 experienced reactive cutaneous capillary endothelial proliferation, compared to 0 patients in group 2. All 59 patients in our cohort had undergone CT before surgery, objective response rates (ORRs) were significant higher in group 1 versus group 2 [17 (89.5%) vs. 18 (45%), P = 0.001; Table 3].

**Table 2:** Incidence of adverse events in the two groups

Adverse Events	Group 1 (n = 19)		Group 2 (n = 40)	
	Any	Grade ≥ 3	Any	Grade ≥ 3
Anaemia, n (%)	2 (10.5)	0	5 (12.5)	2 (5)
Leukopenia, n (%)	6 (31.6)	1(5.3)	14 (35.0)	0
Neutropenia, n (%)	1 (5.3)	0	3 (7.5)	1(2.5)
Thrombocytopenia, n (%)	1 (5.3)	0	3 (7.5)	0
Increased aspartate/alanine aminotransferase, n (%)	1 (5.3)	0	2 (5)	0
Decreased estimated glomerular filtration rate, n (%)	0	0	1 (2.5)	0
Anorexia, n (%)	5 (26.3)	0	9 (22.5)	0
Diarrhoea, n (%)	0	0	2 (5)	0
Constipation, n (%)	1 (5.3)	0	3 (7.5)	0
Reactive cutaneous capillary endothelial proliferation, n (%)	4 (21.1)	0	0	0
Hypothyroidism, n (%)	2 (10.5)	0	1 (2.5)	0

**Table 3:** Responses to neoadjuvant therapy

Response to neoadjuvant therapy	Group 1 (n = 19)	Group 2 (n = 40)	*P* value
ORR	17(89.5)	18(45)	< 0.001
cCR, n (%)	2 (10.5)	0	
cPR, n (%)	15 (78.9)	18 (45.0)	
cSD, n (%)	2 (10.5)	20 (50.0)	
cPD, n (%)	0	2 (5.0)	

ORR, objective response rate; cCR, clinical complete response; cPR, clinical partial response; cSD, clinical stable disease; cPD, clinical progressive disease

## Surgical and postoperative outcomes

The surgical outcomes are summarized in Table 4. The operation time was longer in group 2 than in group 1 (324.6 ± 16.6 min vs. 363.6 ± 11.8 min); however, the difference was only marginally significant (P = 0.064). Blood loss, the number of harvested lymph nodes and the number of positive lymph nodes were comparable between the two groups. The postoperative hospital stay tended to be longer in group 1 [18 (16–21) days vs. 16.5 (13.25–30.50) days], but the difference was not significant (P = 0.358). The postoperative outcomes are summarized in Table 5. In group 1, one (5.3%) patient died within 30 days after surgery due to severe septic shock after anastomotic leakage. In group 2, no deaths occurred within 30 days after surgery, but 2 (5%) patients died within 90 days of surgery from respiratory failure, but there was no significant between-group difference with respect to the incidence of postoperative complications or short-term surgical outcomes.

**Table 4:** Surgical outcomes

Surgical outcomes	Group 1 (n = 19)	Group 2 (n = 40)	*P* value
Operation time (min)	324.6 ± 16.6	363.6 ± 11.8	0.064
Blood loss (mL)	150 (100–200)	100 (100–200)	0.310
Harvested lymph nodes, n	23(20–30)	26 (21–32)	0.276
Positive lymph nodes, n	0(0–1)	0(0-1.75)	0.410
Hospital stay (days)	18(16–21)	16.5(13.25–30.50)	0.358

Data are presented as the mean ± standard deviation or median and interquartile ranges.

**Table 5:** Postoperative outcomes

Postoperative outcome	Group 1 (n = 19)	Group 2 (n = 40)	*P* value
Complications, n (%)			
Atelectasis	0 (0)	2 (5.0)	> 0.999
Pneumonia	4 (21.1)	15 (37.5)	0.206
Respiratory failure	1 (5.3)	3 (7.5)	> 0.999
Chylothorax	0 (0)	2 (5.0)	0.206
Empyema	1 (5.3)	8 (20.0)	> 0.999
Pleural effusion	3 (15.8)	4 (10)	> 0.999
Arrhythmia	2 (10.5)	5 (12.5)	0.279
Anastomotic leakage	1 (5.3)	9 (22.5)	0.832
Recurrent nerve paralysis	1 (5.3)	4 (10.0)	> 0.999
Haematology-related complications	0 (0)	3 (7.5)	0.201
Incision infection	0 (0)	3 (7.5)	0.912
Death in hospital, n (%)	0	0	
30-day mortality, n (%)	1 (5.3)	0	0.322
90-day mortality, n (%)	1 (5.3)	2 (5.0)	> 0.999

## Pathological assessment

The pathological complete response rate in group 1 was significantly higher than that in group 2 [5 (26.3%) vs. 1 (2.5%), P = 0.018; Table 6]. The proportion of patients staged as pT4 was higher in group 2 than in group 1 [6 (31.6%) vs. 18 (45.0%)], but the difference was only marginally significant (P = 0.053). The proportion of patients staged as pN0 was lower in group 2 than in group 1 [13 (68.4%) vs. 20 (50.0%)], but the pathologic N stage was not significantly different between groups (P = 0.179). All patients in group 1 achieved complete resection (R0), while 1 (2.5%) patient in group 2 underwent R1 resection because of high-grade intraepithelial neoplasia in the resection margin of the oesophagus. No significant difference was found in pathological TNM stage between groups.

**Table 6:** Pathological assessment of surgical specimens

Pathological assessment	Group 1 (n = 19)	Group 2 (n = 40)	*P* value
pCR (pT0N0), n (%)	5 (26.3)	1 (2.5)	0.018
Resection margins, n (%)			> 0.999
R0	19 (100)	39 (97.5)	
R1	0	1 (2.5)	
Pathologic T stage, n (%)			0.053
pT0	5(26.3)	1(2.5)	
pT1	4 (21.1)	6 (15.0)	
pT2	2 (10.5)	9 (22.5)	
pT3	2 (10.5)	6 (15.0)	
pT4	6 (31.6)	18 (45.0)	
Pathologic N stage, n (%)			0.179
pN0	13 (68.4)	20 (50)	
pN1	3 (15.8)	10 (25)	
pN2	3 (15.8)	7 (17.5)	
pN3	0	3 (7.5)	
Pathologic stage, n (%)			0.892
I	4 (28.6)	10 (25.6)	
II	1 (7.1)	3 (7.7)	
IIIA	1 (7.1)	5 (12.8)	
IIIB	5 (35.7)	11 (28.2)	
IVA	3 (21.4)	10 (25.6)	

pCR, pathologic complete response; R0, no residual tumour tissue; R1, microscopic residual tumour tissue at the resection margin

## Discussion

In this retrospective study, ESCC patients who received neoadjuvant camrelizumab plus chemotherapy showed a higher pCR rate than those who received neoadjuvant chemotherapy alone. The initial results showed a significantly better therapeutic response in patients who received neoadjuvant camrelizumab plus chemotherapy, without any significant increase in the incidence of adverse events. The surgical outcomes and postoperative outcomes were also comparable between the two groups. These results suggest the feasibility, safety, and effectiveness of the neoadjuvant camrelizumab plus chemotherapy regimen followed by surgery in patients with locally advanced ESCC.

Although the differences were not significant, group 1 showed both a higher proportion of patients staged as cT4 [14 (73.7%) vs. 28 (70%)] and a lower proportion of patients staged as cN0 [12 (63.2%) vs. 28 (70%)]. After neoadjuvant therapy and surgery, group 1 showed both a lower proportion of patients staged as pT4 [6 (31.6%) vs. 18 (45.0%)] and a higher proportion of patients staged as pN0 [13 (68.4%) vs. 20 (50.0%)]. These results suggest better tumour regression in response to neoadjuvant camrelizumab plus chemotherapy, even though the differences were not statistically significant. In our study, the pCR rate was significantly higher in group 1 [5 (26.3%) vs. 1 (2.5%), P = 0.018],, and this parameter is an important prognostic factor associated with survival benefits among patients with locally advanced oesophageal carcinoma. In previous studies, the 5-year survival rate of patients who achieved pCR was over 50%, regardless of tumour histology [8, 9]. The pCR rate of patients treated with neoadjuvant chemotherapy combined with immunotherapy in our study was 26.3%, while that in previous studies ranged from 17 to 45.5%. The difference may be attributable to the different neoadjuvant therapy regimens used [10–13]. In our study, patients underwent neoadjuvant chemotherapy according to the results of the JCOG9907 trial [14]. However, the regimen used in our study was based on docetaxel instead of 5-fluorouracil, as taxane-based neoadjuvant chemotherapy might be associated with better response rates and more favourable survival in patients with advanced ESCC [15–17]. Klevebro et al. found that nCRT resulted in a higher histological complete response rate and higher R0 resection rate than nCT [2]. Additionally, in the PALACE-1 trial, chemoradiotherapy combined with pembrolizumab led to a pCR rate as high as 55.6% among patients with locally advanced ESCC [18], but grade III and higher AEs were observed in 13 (65%) patients, compared to only 1 (5.3%) group 1 patient in our study. The optimal neoadjuvant therapy regimen for patients with locally advanced ESCC remains unclear.

In our study, the incidence of treatment-related toxicity was comparable between the two groups. In the study by Kanjanapan et al., nearly half of all clinically significant immune-related adverse events requiring corticosteroids, hormone replacement, immunotherapy delay, or discontinuation of treatment occurred within the first 8 weeks of treatment [19]. However, during the first 2 cycles of neoadjuvant immunochemotherapy in our study (approximately 8 weeks), no serious immune-related adverse events occurred, and the toxicity effects were treatable and reversible. Moreover, no increase in postoperative complications was observed with neoadjuvant immunochemotherapy. Pneumonia was the most frequent postoperative complication in both groups [4 (21.1%) vs. 15 (37.5%), P = 0.206]. As immune-related adverse events typically have a delayed onset and prolonged duration, postoperative immune-related pneumonia should not be overlooked. Zhao et al. reported the occurrence of immune-related pneumonia one week after surgery in a patient with locally advanced non-small cell lung cancer who underwent neoadjuvant immunotherapy; the patient recovered with timely intervention with corticosteroids [20]. However, few studies have reported postoperative immune-related pneumonia in patients with ESCC and similarly, none of our patients experienced immune-related pneumonia. This suggests a relatively low incidence of immune-related pneumonia in patients with ESCC. Further studies are required to determine whether the low rate of occurrence is related to the immune microenvironment in ESCC.

The status of the immune tumour microenvironment is classified into four types based on programmed death ligand-1 (PD-L1) expression and the density of tumour infiltrating lymphocytes (TILs) [21]. PD-L1 is expressed by tumour cells, helps these cells to evade the host immune response and is recognized as a poor prognostic biomarker for patient survival and a positive predictive biomarker for the efficacy of ICIs [22, 23]. Nevertheless, ICIs may not be effective in the absence of intratumoral infiltration of lymphocytes. The type III tumour microenvironment, defined as PD-L1 expression positivity and lack of T-cell infiltration, is the dominant type in ESCC (66.1%), indicating the need for an approach that comprises a combination of ICIs with promotion of cell infiltration in tumours [24]. In the KEYNOTE-590 trial, chemotherapy plus pembrolizumab was found to be superior to chemotherapy alone in terms of OS in patients with ESCC [median 12.6 vs. 9.8 months; hazard ratio (HR) 0.72; 95% CI 0.60–0.88; P = 0.0006] [6]. The cytotoxic effects of chemotherapy can lead to the death of cancer cells and promote the release of tumour antigens into the local microenvironment, which induces the infiltration of T lymphocytes and exerts their immune effects [24, 25]. Previous studies have shown a significant increase in PD-L1 expression and CD8 TIL density after both neoadjuvant chemotherapy and neoadjuvant chemoradiotherapy in ESCC [22, 26]. These findings implied that neoadjuvant therapy with chemotherapy and ICIs may elicit a better antitumour effect than ICIs alone. The results of our study showed that neoadjuvant immunochemotherapy had promising clinical benefits with a manageable safety profile, which is consistent with previous studies [27, 28], suggesting that the combination of neoadjuvant chemotherapy with immunotherapy may be a breakthrough for patients with locally advanced ESCC.

Some limitations of our study should be taken into account. First, this was a retrospective study. Group 1 showed both a higher proportion of cT4 disease and a lower proportion of cN0 disease, which implied that this group had more progressive tumours than group 2, even though the differences were not statistically significant. In addition, some patients may experience tumour progression after neoadjuvant therapies and lose the opportunity to undergo surgery. Thus, our results may potentially have been influenced by selection bias. Second, the pathological tumour regression grade and PD-L1 status were unclear. As a retrospective study, we collected pathological data from pathological examination reports, but unfortunately some reports did not include a description of the pathological tumour regression grade. PD-L1 status was not detected before and after neoadjuvant therapy, and whether the pathological tumour regression grade or expression of PD-L1 would have an impact on postoperative adjuvant treatment or helping predict prognosis could not be evaluated. Third, the sample size of the study was small. In China, camrelizumab has not been approved as a neoadjuvant regimen until now. Besides it was very expensive before March 2021, only a small number of our patients could afford it. To overcome the limitations of this study, more multicentre, prospective randomized controlled trials are required in the future. Overall, our study provides primary evidence of the safety and efficacy of neoadjuvant immunochemotherapy in patients with ESCC.

## Conclusion

Neoadjuvant chemotherapy plus camrelizumab followed by surgery may be associated with a promising pCR rate and a manageable safety profile for patients with locally advanced ESCC.

## Data Availability

The data used to support the findings of this study are available from the corresponding author upon reasonable request.

## Abbreviations

ESCC: oesophageal squamous cell carcinoma
PD-1: programmed death-1
pCR: pathological complete response
OS: overall survival
nCRT: neoadjuvant chemoradiotherapy
nCT: neoadjuvant chemotherapy
ICIs: immune checkpoint inhibitors
CT: computed tomography
MRI: magnetic resonance imaging
ECT: emission computed tomography
PET: positron emission tomography
RECIST: Response Evaluation Criteria In Solid Tumors
PD-L1: programmed death ligand-1
TILs: tumour infiltrating lymphocytes
